# Improving Si solar cell performance using Mn:ZnSe quantum dot-doped PLMA thin film

**DOI:** 10.1186/1556-276X-8-291

**Published:** 2013-06-20

**Authors:** Dan-Chen Cheng, Hong-Chen Hao, Miao Zhang, Wei Shi, Ming Lu

**Affiliations:** 1 Department of Optical Science and Engineering, and Shanghai Ultra-Precision Optical Manufacturing Engineering Center, Fudan University, Shanghai 200433, China; 2 Materials Physics, Royal Institute of Technology KTH, Kista 164 40, Sweden

**Keywords:** Si solar cell, Quantum dots, Photoluminescence conversion, Antireflectin, 78.55.-m, 84.60.Jt

## Abstract

Poly(lauryl methacrylate) (PLMA) thin film doped with Mn:ZnSe quantum dots (QDs) was spin-deposited on the front surface of Si solar cell for enhancing the solar cell efficiency via photoluminescence (PL) conversion. Significant solar cell efficiency enhancements (approximately 5% to 10%) under all-solar-spectrum (AM0) condition were observed after QD-doped PLMA coatings. Furthermore, the real contribution of the PL conversion was precisely assessed by investigating the photovoltaic responses of the QD-doped PLMA to monochromatic and AM0 light sources as functions of QD concentration, combined with reflectance and external quantum efficiency measurements. At a QD concentration of 1.6 mg/ml for example, among the efficiency enhancement of 5.96%, about 1.04% was due to the PL conversion, and the rest came from antireflection. Our work indicates that for the practical use of PL conversion in solar cell performance improvement, cautions are to be taken, as the achieved efficiency enhancement might not be wholly due to the PL conversion.

## Background

Commercial solar cells employ only a small portion of the solar spectrum for photoelectric conversion, with the available wavelengths covering the visible to near-infrared (NIR) regimes [1]. To fully use the solar emission energy, various light frequency-conversion approaches have been proposed [2-17], which convert IR or ultraviolet (UV) lights into visible ones, the so called up- and down-conversions, respectively. So far, the photoluminescence (PL) conversion, as a type of down-conversion, seems more potentially available in solar cell efficiency enhancement. However, its practical use is actually uncertain, as other factors such as antireflection (AR) might also contribute to the efficiency enhancement in addition to the PL conversion, making the assessment of real contribution from PL conversion doubtful [6,9-14]. Although in our recent work [10], we have noticed this problem and tried to single out the contribution of PL conversion, systematic studies and convincing experimental facts are still lacking. This work aims to solve the puzzling problem by offering a combined approach and evaluating how important on earth the PL conversion could be in improving solar cell efficiency. We selected a material with high PL conversion efficiency (> 40%), i.e., Mn-doped ZnSe quantum dots (Mn:ZnSe QDs). The QDs were dispersed within an organic solution, poly(lauryl methacrylate) (PLMA), which was then spin-coated on the front surface of commercial Si solar cell. The photovoltaic (PV) responses to monochromatic and AM0 light sources were investigated, combined with reflectance and external quantum efficiency (EQE) measurements. With these, the real contribution from PL conversion to the solar cell efficiency enhancement was unambiguously identified and assessed.

## Methods

**Figure 1:**
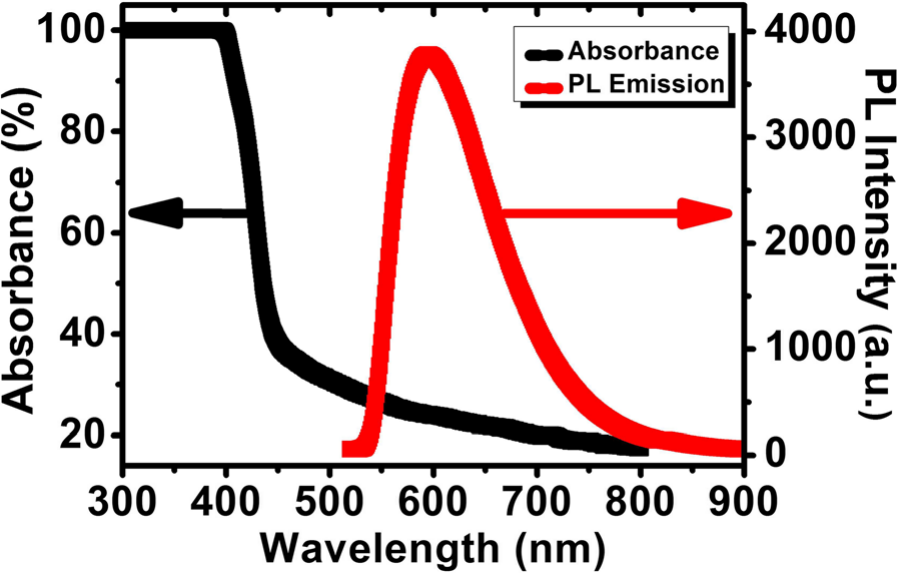
Absorption and PL emission spectra of Mn:ZnSe QDs.

**Figure 2:**
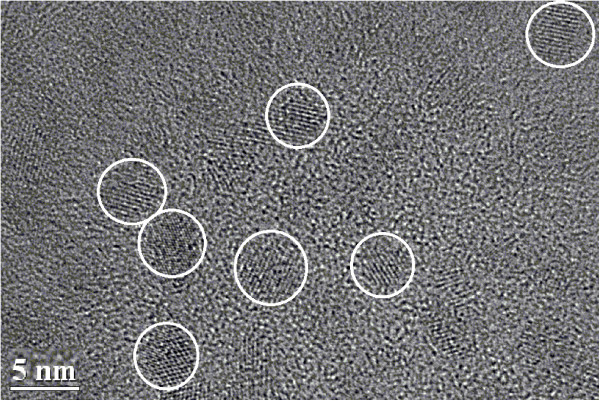
TEM image of the Mn:ZnSe QD distribution.

Mn:ZnSe QDs immersed within toluene were purchased from ZKWY Biotech Incorporation Ltd., Beijing, China. Figure 1 shows their absorption and PL spectra, which reveal the feature of PL conversion from UV/blue to orange/red regimes. The PL efficiency is > 40%. Figure 2 gives a transmission electron microscopy (TEM) image of the QDs dispersed on a Cu grid, acquired with a FEI spectrometer (G2F20, Tecnai, Amsterdam, The Netherlands). The average QD size is 4.8 ± 0.2 nm. Crystalline Si solar cells (20 × 14 mm^2^ in size) without AR treatment were offered by the Shanghai Institute of Space Power Supply, Shanghai, China. The QD suspension was firstly mixed within PLMA (Sigma-Aldrich Co. LLC., St. Louis, MO, USA) and then deposited onto the surface of solar cell with a spin coater. QD concentration (*C*_QD_) was determined by adjusting the proportions of QD suspension and PLMA. The thickness of QD-doped PLMA was around 150 nm as measured using a stylus-profiler (ET3000, Kosaka Laboratory Ltd., Chiyoda-ku, Tokyo, Japan). Reflectance spectra of Si coated with QD-doped PLMA were obtained with an UV–vis-NIR spectrophotometer (UV-3101PC, Shimadzu Corporation, Nakagyo-ku, Kyoto, Japan). PL spectra were recorded on a fluorescence spectrometer (F4500, Hitachi High-Tech, Minato-ku, Tokyo, Japan). Monochromatic lights from one He-Cd laser and other three semiconductor lasers with *λ* = 325, 473, 650, and 980 nm, respectively, were used to investigate the PV responses of short-circuit current (*I*_SC_). Also, a simulated all-solar-spectrum (AM0) PV response was measured on a solar simulator (94023A, Newport Corporation, CA, USA) to acquire the PV parameters of photoelectric conversion efficiency (*η*), fill factor (FF), *I*_SC_, and open-circuit voltage (*U*_OC_). The EQE measurement of solar cell was performed on a QE/IPCE system of Oriel/Newport.

## Results and discussion

**Figure 3:**
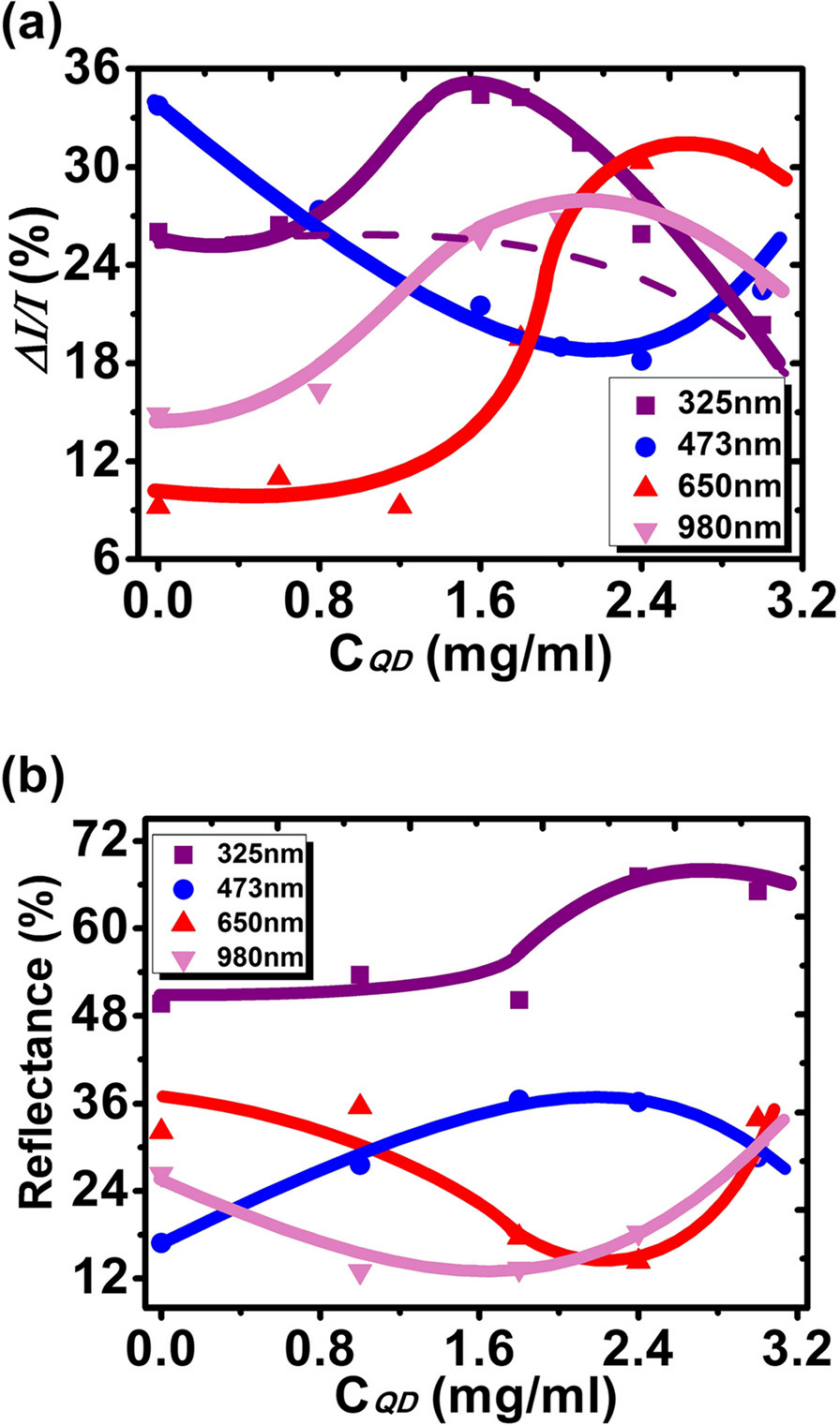
**Short-circuit current enhancements (a) and reflectance coefficients (b) vs QD concentration (*C***_**QD**_**) for four monochromatic light sources.**

Figure 3a shows short-circuit current enhancements (Δ*I*/*I*’s) under illuminations of four monochromatic light sources (*λ* = 325, 473, 650, and 980 nm) as functions of C_QD_. Δ*I*/*I* is defined as (*I*_1_−*I*_bare_)/*I*_bare_, where *I*_bare_ and *I*_1_ are *I*_SC_’s for bare Si solar cell and Si solar cell coated with QD-doped PLMA, respectively. Figure 3b gives the corresponding trends of reflectance for the four wavelengths. It is seen that except for that of UV (*λ* = 325 nm), the Δ*I*/*I* trends of other three wavelengths can be well explained in terms of their reflectance ones. For instance, for the blue (*λ* = 473 nm) response, with the increasing *C*_QD_, the reflectance increases steadily, and after reaching the maximum around 2.2 mg/ml, it starts to decrease. Due to this evolution of reflectance, Δ*I*/*I* decreases steadily at first, and at around 2.2 mg/ml, it starts to increase. As compared to the blue, red (*λ* = 650 nm), and NIR (*λ* = 980 nm) ones, the UV response looks like ‘abnormal’; it does not decrease monotonously in terms of the trend of reflectance but shows a raised structure peaking around 1.6 mg/ml. The appearance of such a raised structure should be due to the PL conversion under UV illumination. Since the absorption edge of QDs as indicated in Figure 1 is approximately 450 nm, it is thus concluded that the PL conversion takes place at wavelengths less than approximately 450 nm. Since the current increase trend correlates monotonously with that of reflectance when the PL conversion does not happen as the cases of *λ* = 473, 650, and 980 nm, for the case of UV in Figure 3a, the contribution of *pure* AR to Δ*I*/*I* could then be represented by a monotonously changing curve as indicated by the dashed line, which was drawn through extrapolating the data at *C*_QD_ < approximately 0.8 mg/ml and *C*_*QD*_ > approximately 2.8 mg/ml, where the PL conversion contribution was little. Therefore, at *C*_QD_ = 1.6 mg/ml, Δ*I*/*I* reads 35.07%, among which, approximately 9.66% is from the effect of PL conversion as calculated, and the rest approximately 25.41% due to AR. In the following, we will focus on the cases of *C*_QD_ = 0 and 1.6 mg/ml only and assess the contribution of PL conversion to Si solar cell efficiency enhancement under two AM0 conditions.

**Figure 4:**
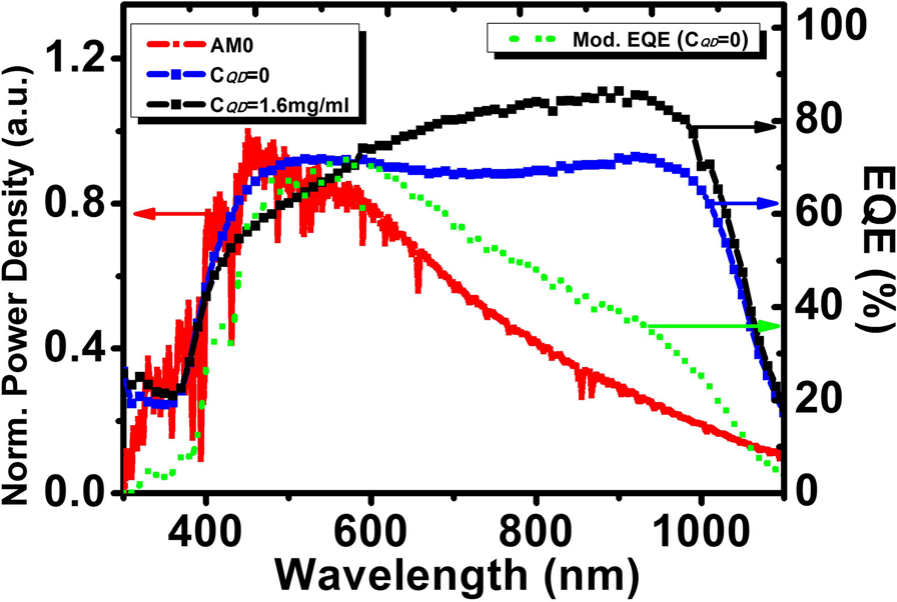
**EQE curves and emission spectrum of the standard AM0.** EQE curves for Si solar cells coated with QD-doped PLMA with *C*_QD_ = 0 and 1.6 mg/ml (right ordinate) and the power-density-normalized standard AM0 spectrum (left ordinate). The dotted curve is the modified EQE curve for *C*_QD_ = 0 (right ordinate) under the AM0 condition.

**Figure 5:**
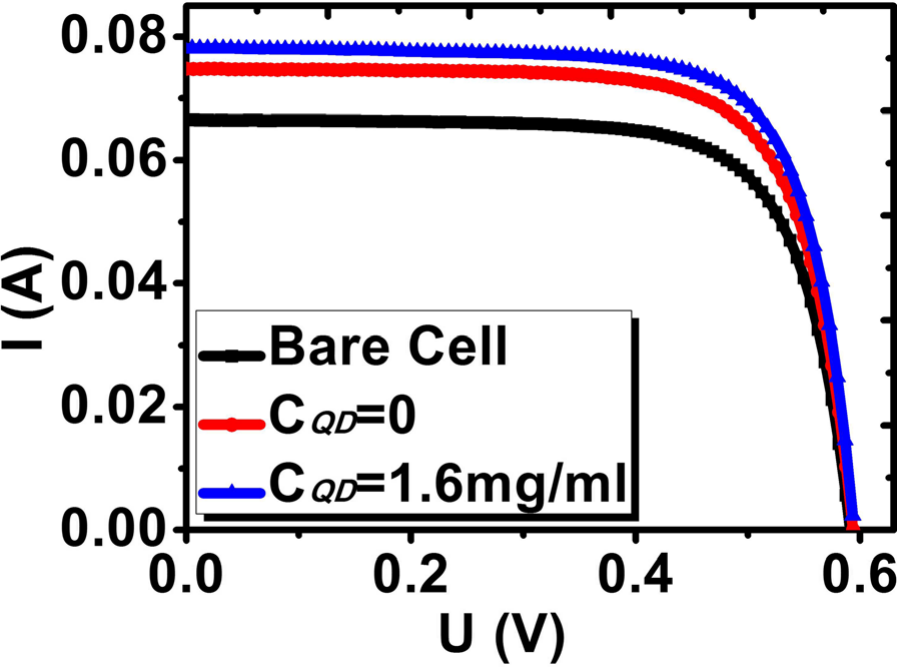
***I-V *curves.** For bare Si solar cell and Si solar cells coated with QD-doped PLMA at *C*_QD_ = 0 and 1.6 mg/ml.

Figure 4 gives the measured EQE curves for Si solar cells with *C*_QD_ = 0 and 1.6 mg/ml (right ordinate), together with the emission spectra of a standard AM0 [18] (left ordinate). A solar cell efficiency enhancement is defined as Δ*η*/*η*_0_ = *(η*_1_*− η*_0_*)*/*η*_0_, where *η*_0_ and *η*_1_ are photoelectric conversion efficiencies of Si solar cell coated with QD-doped PLMA with *C*_QD_ = 0 and *C*_QD_ ≠ 0, respectively. It should be noted here that unlike Δ*I*/*I,* which is with respect to bare Si solar cell, Δ*η*/*η*_0_ is with respect to Si solar cell coated with pure PLMA (*C*_QD_ = 0). In Table 1, the measured and calculated PV parameters for different solar cells are listed. Based on Figure 4, Δ*η*/*η*_0_ could be calculated as follows. The AM0 intensity times EQE yields the modified EQE curve. An example is illustrated in Figure 4 by the dotted curve for AM0 × EQE at *C*_QD_ = 0. The modified EQE curve gives the efficiency response for each wavelength in AM0 spectrum. The summation of all the responses, i.e., the area under the modified EQE curve may represent the solar cell efficiency. Δ*η*/*η*_0_ can thus be calculated as the area difference between *C*_QD_ = 1.6 mg/ml and 0, divided by the area for *C*_QD_ = 0. The calculated Δ*η*/*η*_0_ was 5.96%, which is close to the measured value. For *λ* < approximately 450 nm, the efficiency enhancement could now be regarded as wholly from the contribution of PL conversion, since the reflectance coefficients at *C*_QD_ = 0 and 1.6 mg/ml are nearly the same as shown in Figure 3b. Hence, the PL contribution was calculated as the area difference between *C*_QD_ = 1.6 mg/ml and 0 for *λ* < approximately 450 nm only, divided by the whole area for *C*_QD_ = 0. It was 1.04%. Therefore, the rest 5.96% − 1.04% = 4.92% was due to AR. In Figure 5, *I-V* curves for bare Si solar cell and Si solar cell coated with QD-doped PLMA (*C*_QD_ = 0 and 1.6 mg/ml) are depicted. *U*_OC_ and FF change slightly; only the *I*_SC_ varies steadily, leading to a change in *η*. In Table 1, Δ*η*/*η*_0_ for *C*_QD_ = 3.0 mg/ml is as high as 9.97%, which is the highest efficiency enhancement achieved in this work. However, from Figure 3a, it is certain that the PL contribution to Δ*η*/*η*_0_ at *C*_QD_ = 3.0 mg/ml is very little. The AR effect contributes dominantly, which could be attributed to the modification of refractive index gradient [19]. Since many other efficient AR approaches have been developed [19-22], the effect of AR will not be further discussed here.

**Table 1:** PV parameters for Si solar cells after treatments

**Sample**	***I***_**SC **_**(mA)**	***U***_**OC **_**(V)**	**FF (%)**	***η *(%)**	**Δ *η */*η***_**0 **_**(%)**	**Δ *η */*η***_**0 **_**(%) (calculated)**
Bare cell	66.50	0.59	73.65	11.12	-	-
*C*_QD_ = 0	74.74	0.59	73.78	12.54	0.00	0.00
*C*_QD_ = 1.6 mg/ml	78.10	0.59	74.38	13.24	5.58	5.96
*C*_QD_ = 3.0 mg/ml	81.08	0.60	74.50	13.79	9.97	-

In this work, AM0 solar simulator rather than the more conventional AM1.5 one has been used. This is because the effect of PL conversion on the performance improvement of solar cell is more applicable in the environment with higher UV proportions. The UV proportion in the high altitude or outer space environment, which the AM0 condition mimics, is generally two to three times that in the normal AM1.5 one. On the other hand, from Figure 4, it is seen that the solar cell has high EQE in a broad wavelength range of approximately 450 to 1,000 nm; therefore, although for each wavelength, the corresponding reflectance changes with the changing film thickness due to the light interference, the overall efficiency enhancement is not sensitive to the film thickness, as what we found in our experiments for the film thickness in the range of 100 to 300 nm. However, it should be pointed out that to optimize the effect of PL conversion, the film thickness could be a concern in the work that follows; meanwhile, the side edges of PLMA film would be coated with SiO_2_ by a simple mask method so as to prevent visible lights due to the PL conversion escaping from the side areas and let them be absorbed by the solar cell underneath.

## Conclusions

In this work, PLMA thin film doped with Mn:ZnSe QDs was spin-deposited on the front surface of Si solar cell in order to improve the solar cell efficiency via PL conversion. Significant efficiency enhancements (approximately 5% to 10%) were achieved indeed under AM0 conditions. Both the effects of AR and PL conversion contributed to the solar cell efficiency enhancements but that of PL took a small portion. A precise assessment of PL contribution to the efficiency enhancement was made by investigating the PV responses of Si solar cells coated with QD-doped PLMA to monochromatic and AM0 light sources as functions of QD concentration, combined with reflectance and EQE measurements. Our work shows that the real PL contribution might not be all that as reflected by the apparent efficiency enhancement, and cautions are to be taken when applying the PL conversion in this aspect. On the other hand, it indicates again that for practical use of PL conversion, high altitude or/and outer space environments are preferred where the UV proportion is high, and continuing to search for high PL efficiency materials and design efficient optical-coupling structures is still necessary.

## Competing interests

The authors declare that they have no competing interests.

## Authors’ contributions

DCC prepared all the samples and measured the absorbance, PL, short circuit, and *I-V* data. HCH measured the EQE data. MZ helped to prepare samples. WS measured the reflectance data. ML designed the experiments and wrote the manuscript. All authors read and approved the final manuscript.
